# Gut Mucosal Microbiome of Patients With Low-Grade Adenomatous Bowel Polyps

**DOI:** 10.1016/j.gastha.2025.100687

**Published:** 2025-04-28

**Authors:** Zoe Welham, Jun Li, Benita Tse, Alexander Engel, Mark P. Molloy

**Affiliations:** 1 Bowel Cancer and Biomarker Laboratory, Kolling Institute, School of Medical Sciences, The University of Sydney, St. Leonards, Australia; 2 Colorectal Surgical Unit, Royal North Shore Hospital, St. Leonards, Australia; 3 Sydney Medical School, The University of Sydney, Sydney, Australia

**Keywords:** Adenoma, Bowel Neoplasia, Microbiome, Colorectal, 16S rRNA

## Abstract

**Background and Aims:**

Colorectal cancer etiology is multifactorial and influenced by colonic environmental exposures leading to the accumulation of genetic lesions in precancerous polyps. There is growing recognition for a role of the gut microbiome in colorectal cancer progression, but the structure of the gut mucosal microbiome in the early stages of polyp growth is limited. The aim of this study was to characterize the gut mucosal microbiome from patients with low-grade conventional bowel neoplasia compared to symptomatic but polyp-free patients.

**Methods:**

In this case-control study conducted at a tertiary referral hospital, 148 symptomatic patients undergoing colonoscopy were prospectively recruited. Mucosal biopsies adjacent to low-grade dysplasia (LGD) adenomatous polyps were used for 16S rRNA gene amplicon sequencing to define bacterial taxonomies relative to polyp-free controls.

**Results:**

Minimal differences in gut mucosa community diversity measures were observed between participants with or without LGD adenomas. After correcting for clinical covariates, patients with adenomas in the proximal colon revealed elevated amplicons from *Parabacteroides distasonis, Bacteroides uniformis,* and unassigned *Lachnospiraceae spp*. *Bacteroides/Phocaeicola massiliensis* was the only microbe consistently found to be decreased in the gut mucosa of LGD adenoma patients compared with controls. Participants with LGD polyps in the distal colon showed more amplicons from *Howardella sp.* and *Blautia faecicola*.

**Conclusion:**

This study identified microbial candidates in the colonic mucosa that are associated with adenomatous LGD bowel neoplasia as an early step in the colorectal carcinogenesis pathway.

## Introduction

Worldwide, colorectal cancers (CRCs) account for 10% of cancer incidence and second highest for cancer-related mortality.^1^ CRCs develop from benign precursor polyp lesions that temporally accumulate genetic mutations favorable for cell proliferation and transformation into adenocarcinomas. It is increasingly clear that the gut microbiome contributes to the health and homeostasis of the colon and that alterations to the microbial community and outgrowth of specific pathogens can facilitate CRC tumorigenesis.^2^ The human bowel hosts a diverse community of more than 1000 species of bacteria, along with viruses, archaea, and fungi.^3^^,^^4^ It is often referred to as “the second genome” due to its role as a dynamic contributor to host health and disease in energy homeostasis,^5^ metabolism,^6^ and immunology.^7^ The gut microbiota may be beneficial, neutral, or pathogenic, depending on their location, the metabolites they produce, their regulated abundances due to interactions with other species, and the host’s biological processes.

Evidence of the importance of the gut microbiome in CRC development comes from mouse studies that administered stool samples from CRC patients to germ-free and conventional mice treated with the carcinogen azoxymethane, which resulted in increased polyp number, intestinal dysplasia and proliferation, increased inflammation markers, and increased Th1 and Th17 cells compared to mice exposed to stool from healthy individuals.^8^

Investigating the specific bacteria that may contribute to the early stages of CRC progression is an active branch of research: several studies have sequenced different regions of the 16S rRNA gene from CRC patient stool samples as a convenient proxy for the gut environment. These studies report few similarities in the differentially abundant bacteria observed between adenoma cases and healthy controls, potentially because of technical as well as biological reasons. For example, studies differ in the polymerase chain reaction primers used to generate 16S rRNA amplicons, and the use of different bioinformatics processing pipelines leads to interstudy variances in bacterial amplification and taxonomy assignment.^9^^,^^10^ Moreover, individual participant variation due to ethnicity, diet, and lifestyle greatly influences microbial composition, potentially masking microbial changes induced by cancer development.

Despite these challenges, a few studies have consistently indicated that bacteria including *Fusobacterium nucleatum* are associated with some precancerous polyps as well as CRC.^11^, ^12^, ^13^ However, the literature lacks consistency. For example, Goedert *et al* reported a trend toward depletion of bacteria from *Fusobacterium* in feces of adenoma patients compared to healthy controls.^14^ While stool samples have some advantages in being noninvasive to collect and have diagnostic potential, the bowel mucosa microbial composition differs significantly from luminal microbiota quantified in stool,^15^, ^16^, ^17^, ^18^ and the stool microbiome has been characterized as only partially similar to the mucosal microbiome in CRC.^19^ Although sampling is more complicated, gut mucosal biopsies may be more informative to build our understanding of microbiota communities associated with colorectal neoplasia.

The literature on the gut mucosal microbiota from patients with bowel polyps is sparse; however, several studies utilizing 16S rRNA gene sequencing to compare adenoma mucosal biopsies with adjacent healthy tissue, or with biopsies from healthy control cases, have reported the enrichment of Proteobacteria, and a relative depletion of Firmicutes in adenomas compared to controls. Select example studies that compared polyp biopsies with adjacent healthy mucosa suggest that polyp tissue shows enrichment in *Lactobacillus*, *Klebsiella*, *Helicobacter*, *Ruminococcus*, *Prevotella*, *Pseudobutyrivibrio*, *Alistipes,*^20^ and *Clostridium XIVa sp*^21^ with a depletion in *Bifidobacterium*, *Faecalibacterium*, *Escherichia Shigella*, *Bacteroides*, *Coprococcus*, *Erysipelatoclostridium*, *Blautia*, *Propionibacterium*, *Collinsella*, *Romboutsia*, *Ruminococcus, Lachnoclostridium*, *Dorea*, *Anaerostipes,*^20^ and *Faecalibacterium sp*.^21^ compared to healthy adjacent tissue.

When comparing mucosal biopsies from patients with bowel polyps to those without polyps, studies reported enrichment of *Lactococcus*,^22^ *Pseudomonas*,^22^, ^23^, ^24^ *Helicobacter*, *Acinetobacter*,^23^ *Escherichia-Shigella*, *Prevotella*,^25^ *Enterococcus*, *Oscillibacter*, *Mogibacterium,*^26^ and *Ruminococcus gnavus*,^27^ and depleted *Enterococcus*, *Bacillus*,^22^^,^^25^ *Staphylococcus*,^24^^,^^25^ *Blautia*,^27^ *Betaproteobacteriales*, *Klebsiella*, *Burkholderiaceae*, *Subdoligranulum*, *Eubacterium eligens group*, unclassified *Veillonellaceae*, *Lachnospiraceae FCS020 group*, *Lautropia*^26^ *Gemmiger*, and *Bifidobacterium*.^24^

A further complication to unraveling microbial dysbiosis and CRC relates to the observation that proximal and distal tumors often harbor different microbial communities.^28^ However, only limited reports have examined gut microbiomes between left- or right-sided adenomas. *Fusobacterium* was found to be more common in stool from proximally located CRC^29^^,^^30^ and *Blautia, Eryspelotrichales*, *Holdemanella*, *Faecalibacterium*, *Subdoligranulum,* and *Dorea* more abundant in stool from distal CRC.^29^ Other studies have shown that polyp location and histology are important factors associated with microbial composition, and this can be lost in analysis strategies that fail to consider these as covariates.^31^

The objective of this study was to characterize the gut mucosal microbiome adjacent to LGD adenomatous polyps compared to gut mucosa from symptomatic but polyp-free patients, as these lesions represent early steps in CRC carcinogenesis. We prospectively recruited colonoscopy patients and considered covariates of age, sex, body mass index (BMI), and polyp location when comparing gut microbiomes. We applied 4 bioinformatic models to detect differences in microbiota between cases and controls.

## Methods and Materials

### Ethics Statement

This case-control study was conducted in accordance with the Helsinki Declaration, involving participants undergoing scheduled colonoscopy at the tertiary referral hospital Royal North Shore Hospital, Sydney, Australia, from 2020 to 2023. Human research ethics (2019/ETH00301) and governance (2019/STE10535) approval was obtained from the Northern Sydney Local Health District. Written informed consent was obtained from all participants. All study samples and information collected were deidentified by assigning a unique study identity code. We followed the STROBE guidelines for reporting.

### Participant Inclusion and Exclusion Criteria

The study included adults ≥18 years old undergoing scheduled colonoscopy for symptoms including a positive fecal occult blood test, perirectal bleeding, abnormal gut symptoms (including abnormal findings on computed tomography scan, patient-reported gastrointestinal pain, or changes in patient bowel habits exceeding 14 days), or for surveillance due to positive personal or family history of sporadic bowel neoplasia.

The study excluded patients diagnosed with hereditary bowel polyposis diseases, patients with Crohn’s disease, ulcerative colitis, active diverticulitis, or other inflammatory bowel disease, and patients who had taken antibiotics within 4 weeks before the colonoscopy.

### Mucosal Specimen Collection and Assessment

Colorectal polyps were excised following standard clinical care guidelines. For each polyp removed, a 2 mm^3^ mucosal biopsy 20 mm proximal to the polyp was collected for microbiome analysis. Mucosal biopsy strategy was unselected; ie, the first polyp encountered during the colonoscopy was removed and a research mucosal biopsy of adjacent tissue was taken. A maximum of 2 different biopsies were taken if polyps were found at discrete locations. If no colon polyps were detected, 2 random mucosal biopsies were taken in the proximal and distal colon to form the control group. The proximal biopsy was taken within 15 cm of the ileocecal valve, and the distal biopsy was taken when remaining intracolonic scope length was between 25 and 45 cm. All biopsies were placed in a tube and immediately immersed in dry ice, then stored at −80 °C. Each associated polypectomy specimen underwent routine histopathology assessment by a certified pathologist, with findings reported synoptically according to the Royal College of Pathologists of Australasia,^32^ which included polyp histology type, grade, and size.

### Cohort Design

The case cohort for microbiome sequencing was constructed from consecutive consented patients over the period 2020–2023 who had a pathology specimen diagnosis of LGD tubular adenoma or tubulovillous adenoma and whose DNA yielded sufficient quality to pass sequencing QC requirements. The control cohort was assembled from patients who underwent colonoscopy during this period with no polyps detected, with matching to ensure no significant difference in average age, sex, or BMI. Wilcoxon rank sum test was used to compare variables with *P* < .05 considered significant.

### DNA Extraction

Biopsies were rinsed with phosphate-buffered saline prior to lysis. DNA was extracted from mucosal biopsies using the Invitrogen PureLink Microbiome DNA Purification kit (Thermo Fisher Scientific, Waltham, MA, USA, Catalog Number: A29790) according to the manufacturer’s instructions. In summary, specimens were added to 800 μL of lysis buffer and 100 μL of optimization buffer to lyse cells. Samples were briefly vortex mixed then underwent heating at 65 °C for 10 minutes, and bead beating at 50 Hz for 10 minutes. After centrifugation, the solution containing DNA was transferred to a new tube. 900 μL of binding buffer was added to each tube, vortex mixed, then transferred to a kit column. After centrifugation to remove the buffer, DNA bound to the column was washed with 500 μL of ethanol solution. After centrifugation and airing the tubes for 1 minute to remove the ethanol solution, the bound DNA was eluted with 50 μL of Tris buffer, pH 8.0. All centrifugation steps occurred for 1 minute at 14,800 RPM and at 20 °C. All extracted DNA was stored at −80 °C.

### 16S rRNA Gene Sequencing and Bioinformatic Analyses

The V3–V4 region of the 16S rRNA gene was sequenced at the Australian Genome Research Facility using the primer pair 341F CCTAYGGGRBGCASCAG and 806R GGACTACNNGGGTATCTAAT. Paired-end amplicon sequencing (2 × 300bp) was performed using the Illumina MiSeq platform (Illumina, San Diego, CA, USA).

The resulting data underwent a quality check using FastQC (version 0.11.9)^33^ and bioinformatics processing using QIIME2.^34^ DADA2 (version 2022.8)^35^ was used to trim the paired-end reads of the adapter and primer sequences and all reads were truncated to lengths 270 (forward reads) and 205 (reverse reads) to minimize the effect of low-quality reads. The paired-end reads were merged and filtered of phiX and chimeric sequences. Reads that shared greater than 97% identity with human sequences were removed.

All reads were classified to the lowest possible taxonomic rank by using the QIIME2 q2-feature-classifier plugin to train the Naive Bayes classifier, using the Silva v138 99% OTUs full-length sequences database (https://www.arb-silva.de/documentation/release-138/). The data underwent further taxonomic identification using the Genome Taxonomy Database release 214 (https://gtdb.ecogenomic.org/) and the RefSeq database version 16 compiled on 06/11/2020 (https://zenodo.org/records/4735821). The identification with the most specific taxonomic resolution was chosen from the 3 databases by an iterative joining of the 3 database results. R code was obtained by modifying the DADA2 *assignTaxonomy* script (https://github.com/PacificBiosciences/pb-16Snf/blob/main/scripts/dada2_assign_tax.R). An amplicon sequence variant (ASV) table was constructed showing the read counts of each identified ASV for each sample.

### Statistical Analysis

#### Alpha diversity analyses

Alpha diversity refers to the diversity within a particular area or ecosystem. It can be expressed by both the number of different species present (species richness) and by whether each different species is present in the area in equal numbers (species evenness). Alpha diversity measures were calculated on the raw ASV count tables for the 16S rRNA data using the *boxplot_alpha* function from the R package *microbiome* (version 1.22.0).^36^ Shannon’s index was employed to measure alpha diversity, which incorporates phylogenetic relationships between features to produce a qualitative measure of community richness and evenness within each sample. A higher index for this measure reflects higher diversity. Nonparametric 2-sample Wilcoxon tests, using the *stat_compare_means* function from the R package *ggpubr* (version 0.6.0)^37^ were employed to test for statistically significant differences between the polyp and no-polyp groups.

#### Filtering and normalization

The data were filtered to remove ASVs that were present in less than 5% of total samples, as these ASVs were considered to contribute negligible explanatory power for distinguishing between case and controls. Filtered raw abundances from 16S rRNA gene sequencing were then normalized prior to beta diversity analysis using Total Sum Scaling to correct for technical biases that can occur in sequencing platforms that results in uneven abundances between samples. The differential abundance methods employed Centered Log Ratio normalization within their functions.

#### Beta diversity analyses

Beta diversity measures were used to assess for any overall differences in microbiome community between participants with polyps compared to those without polyps. This measure identifies whether the same species are found between sites or whether each site contains unique species compared to other sites. Beta diversity for Total Sum Scaling-normalized proximal mucosa and distal mucosa data was assessed by computing the Bray-Curtis dissimilarity (BCD) distance using the *vegdist* function from the *vegan* R package (version 2.6-4).^38^ The BCD ranges between 0 and 1, where 0 indicates 2 sites share the same number of each type of species—they are completely similar; and 1, which indicates 2 sites share none of the same types of species—they are completely dissimilar. The resulting BCD distances were then projected onto the first 2 principal coordinates of a principal coordinate analysis, using the *ordinate* function from the R package *Phyloseq* (version 1.44.0).^39^

#### Univariate differential abundance analyses

The R packages *Analysis of Compositions of Microbiomes with Bias Correction 2* (ANCOMBC2, version 2.0.2),^40^ *ANOVA-Like Differential Gene Expression Analysis* (ALDEx2, version 1.30.0),^41^ *Multivariable Association Discovery in Population-scale Meta-omics* (MaAsLin, version 1.16.0)^42^ and *Linear (Lin) Model for Differential Abundance (DA) Analysis of High-dimensional Compositional Data* (LinDA) from *MicrobiomeStat* (version 1.1) were employed to identify differentially abundant ASVs associated with polyp status. For these analyses, ASVs were considered differentially abundant if the effect size was greater than 0.2 for the ALDEx2 analysis, where the effect size was calculated as the ratio of the difference between and the difference within condition values. For the other 3 analyses, ASVs were considered differentially abundant with a *P* value < .05. All methods included age and sex as covariates, and ANCOMBC2, MaAsLin, and LinDA also included BMI covariate to assess for the unique influence of polyp status on the microbiome. We chose to discuss microbes detected in at least 2 of the 4 models.

## Results

### Participant Demographic and Clinical Data

This study analyzed the microbiome from gut mucosal biopsies of 148 participants, consisting of 57 cases (LGD adenomas) and 91 controls (no polyps) (Table A1). For adenoma patients, a mucosal biopsy was collected 20 mm adjacent to the polyp and used for the study, as the polyp specimen itself was reserved exclusively for pathology diagnosis. All case specimens were confirmed by histopathology as LGD, conventional adenomatous neoplasia (40 male, 17 female). The majority were small adenomas (<10 mm), while 26% of specimens were advanced adenomas (size >10 mm or tubulovillous). Normal mucosa biopsies obtained from the non-polyp control cohort were used for matching considering age, sex, BMI, and anatomical location (distal/proximal) (53 male, 38 female). The control participants had no colonoscopic evidence of colorectal neoplasia at the time of specimen collection.

A summary of the demographic and clinical data is shown in Table. The average age for participants with polyps was 68 years old (interquartile range [IQR]: 58–74), and those without polyps, 63 years old (IQR: 52–72). Average BMI for participants with polyps was 25.6 (IQR: 21.6–29.7) and for those without polyps, 25.3 (IQR: 23.3–30.1). The groups were matched to ensure there were no significant differences in age (*P* = .09), sex (*P* = .07), or BMI (*P* = .2).

**Table:** Demographic and Clinical Characteristics of the Participants

Characteristic	No polyp	Polyp	*P* value
Participants	91	57	
Specimens	100	68	
Age	63 (IQR 52–72)	68 (IQR 58–74)	.09^a^
Sex			
Female	42	21	.07^a^
Male	58	47	
BMI	25.3	25.6	.2^a^
Unknown	7	11	
Location			
Proximal	48	39	.2^a^
Distal	52	29	
Adenoma size (mm)			
Large (≥10)		16	.001^a^
Small (<10)		52	
Adenoma type			
Tubular -LGD		56	.001^a^
Tubulovillous - LGD		12	
Indications for colonoscopy			
Perirectal bleeding	39	20	.01^a^
Surveillance: history polyp or CRC	14	24	
Abnormal bowel symptoms^b^	29	15	
Surveillance: first-degree relative polyp or CRC	9	2	
FOBT+	9	7	

FOBT, fecal occult blood test.

a Wilcoxon rank sum test.

b Includes abnormal gastrointestinal computed tomography scan, patient-reported gastrointestinal pain, changes in bowel habits exceeding 14 days.

The majority of patients underwent colonoscopy due to indications of perirectal bleeding (polyp = 20, no polyp = 39), for surveillance due to a personal history (polyp = 24, no polyp = 14), or having a first-degree relative with polyps or CRC (polyp = 2, no polyp = 9). Both groups had similar frequency for fecal occult blood test positivity (polyp = 7, no polyp = 9), and patient-reported abnormal bowel symptoms (polyp = 13, no polyp = 16).

### Microbiome Abundance and Prevalence

**Figure 1:**
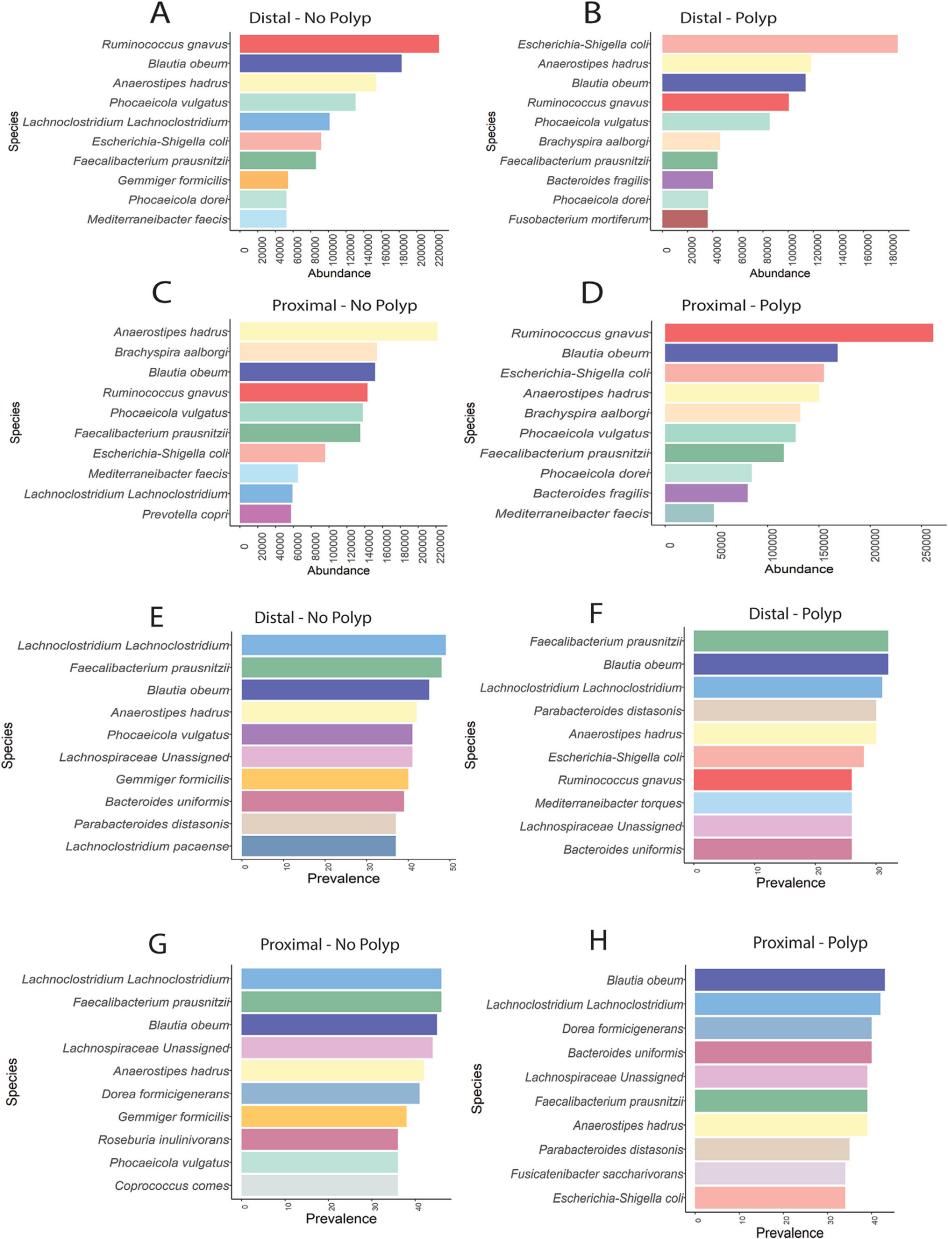
Abundance and prevalence of microbiota at the proximal colon and distal colon. Top 10 microbial species for cumulative abundance (A–D) and prevalence (E–H) in (C, G) distal, no polyps, (D, H) distal, polyps, (A, E) proximal, no polyps, (B, F) proximal, polyps.

16S rRNA reads were used to calculate the abundance and prevalence plots of the bowel mucosal microbiota, dichotomized for colon sidedness (Figure 1).

Based on the top 10 most identified species we noted the distal and proximal colon shared many species, with some of the most abundant being *R gnavus, Escherichia-Shigella coli, Blautia obeum*, and *Anaerostipes hadrus.* In the case group, *E coli* was highly abundant but was observed with reduced counts in the control group. Considering the top 10 species abundance, *Bacteroides fragilis* and *F mortiferum* were most abundant in the polyp case group but not in the non-polyp controls. Conversely, *Gemmiger formicilis, Prevotella copri,* and *Lachnoclostridium spp.* were only found in the top 10 of participants without bowel neoplasia.

Normalized for microbiota prevalence between groups, *E coli* was shown to be highly prevalent in distal and proximal mucosa from adenoma patients only. *R gnavus* and *Mediterraneibacter torques* were high prevalent in distal mucosa from polyp patients, while *Fusicatenibacter saccharivorans* was highly prevalent in proximal mucosa of polyp patients. *G formicillis* was highly prevalent in both distal and proximal mucosa from control patients but not from patients with adenoma. *Coprococcus comes* and *Roseburia inulinivorans* were prevalent species in proximal mucosa from controls, while *Lachnoclostridium pacaense* was prevalent in distal mucosa from control patients.

### Relative Abundance Statistical Analysis

We carried out statistical analysis to compare relative abundances of microbes using family- and genus-level taxonomy for specimens from the distal and proximal colon. Of the most abundant genera (>%2), *Ruminococcus* was the only taxonomy to be more abundant in the proximal colon of adenomatous polyp patients (Table A2), while *Akkermansia* was more abundant in the distal colon of polyp patients (Table A3).

### Microbiome Diversity Analyses

**Figure 2:**
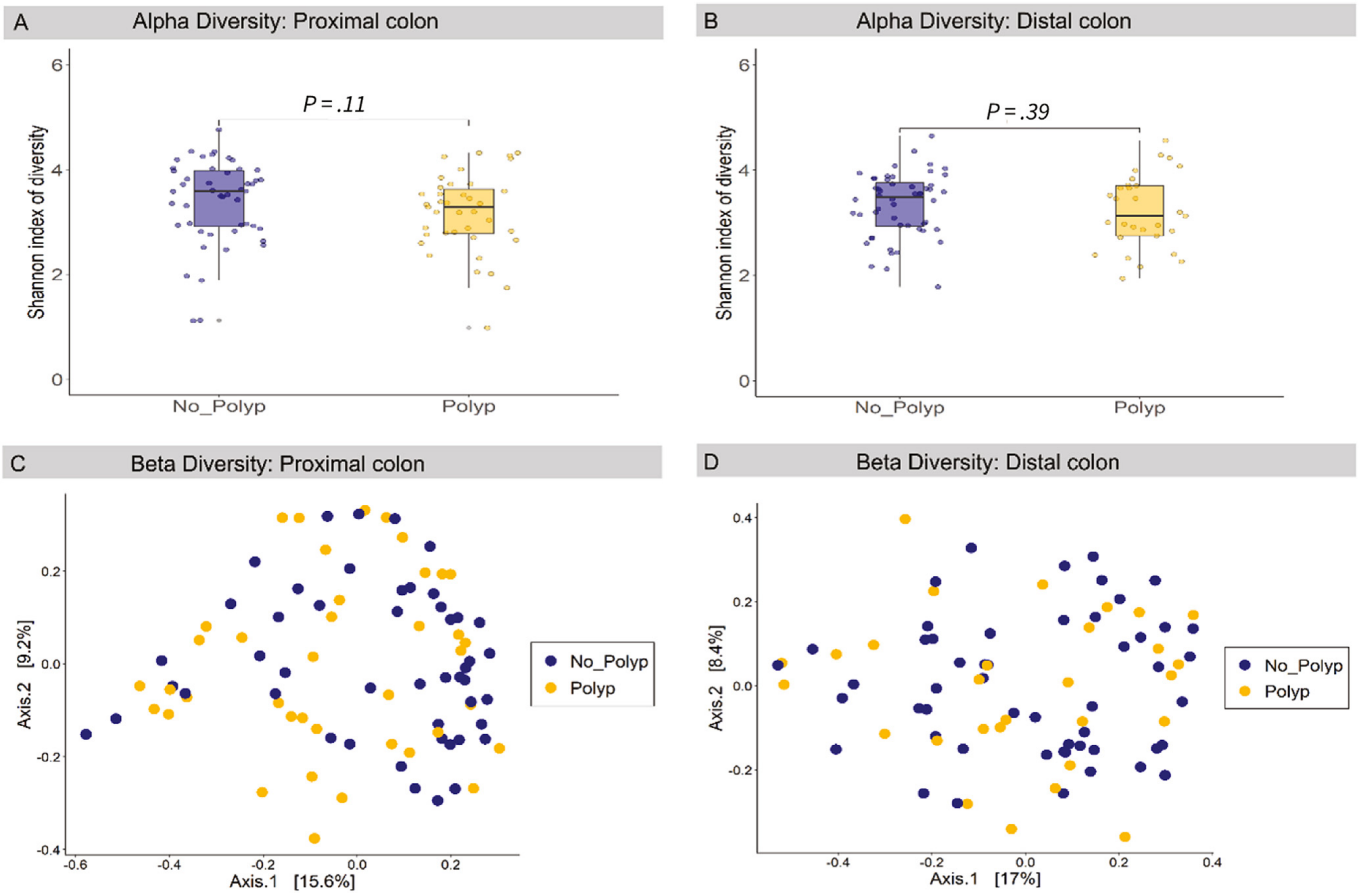
Mucosal microbiome diversity at the proximal and distal colon. Alpha diversity based on Shannon Index (A) proximal colon, (B) distal colon. Beta diversity using principal coordinates analysis to display Bray-Curtis dissimilarity score for the (C) proximal colon, (D) distal colon. Yellow defines polyp specimens, while blue defines control specimens. Wilcoxon *t*-test used to calculate differences in alpha diversity.

Alpha diversity was calculated using Shannon’s Index to assess for species richness and evenness within specimens. Boxplots showing the average scores for patients with adenoma and those without polyps are displayed in Figure 2. Although the mean diversity was lower in the LGD adenoma group, there was no statistically significant difference in species richness or evenness as assessed by a Wilcoxon *t*-test.

Beta diversity for the distal colon and proximal colon was assessed to examine for differences in bacterial community composition between the adenoma and no-polyp groups (Figure 2). The Bray-Curtis dissimilarity scores were displayed by principal coordinates analysis plots, which showed that individual variation was the largest contributor to the variance in microbiome across both colon sides, with no distinct separation of participants with adenomas compared to those without polyps.

### Bioinformatic Models to Assess Differential Abundance in the Proximal Colon Mucosa

**Figure 3:**
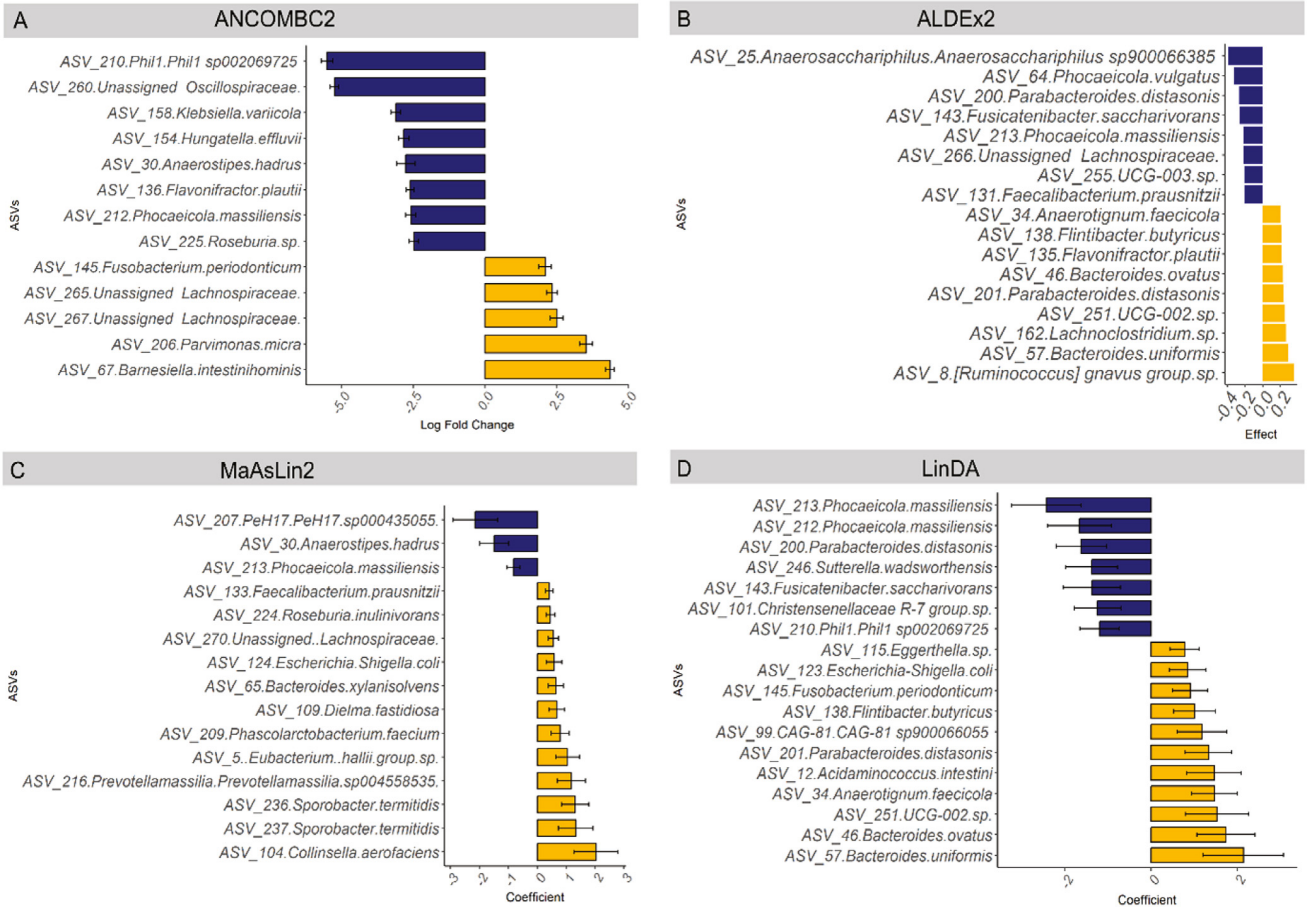
Microbial differential abundance comparing proximal colon mucosa for adenoma and no-polyp participants. (A) ANCOMBC2 analysis (*P* < .05, log fold change >1.5) (B) ALDEx2 (effect size >0.15). (C) MaAsLin2 (*P* < .05). (D) LinDA analysis (*P* < .05). Yellow, elevated in adenoma; blue, decreased in adenoma; black bars indicate standard error. All analyses include age, sex, and BMI as covariates, except for ALDEx2 which includes age and sex. (n = 39 adenoma, 48 control).

We used 4 bioinformatic models incorporating clinical covariates to evaluate differential ASV microbiome abundance. Figure 3 shows bar plots of the ANCOMBC2 (*P* value < .05, log fold change >1.5), ALDEx2 (effect >0.20), MaAsLin2 (*P* value < .05), and LinDA (*P* value < .05) analyses, all of which included age, sex, and BMI as covariates, except for ALDEx2 which included age and sex only. Interestingly, each model reported at least 10 differentially abundant ASVs and there was considerable variation amongst the models. Bacteria that were significantly elevated in participants with adenomas and reported by multiple models were *Bacteroides uniformis, Parabacteroides distasonis,* and unassigned *Lachnospiraceae spp. Bacteroides/Phocaeicola massiliensis* was the only microbe consistently found to be decreased in adenoma patients compared with controls.

### Bioinformatic Models to Assess Differential Abundance in the Distal Colon Mucosa

**Figure 4:**
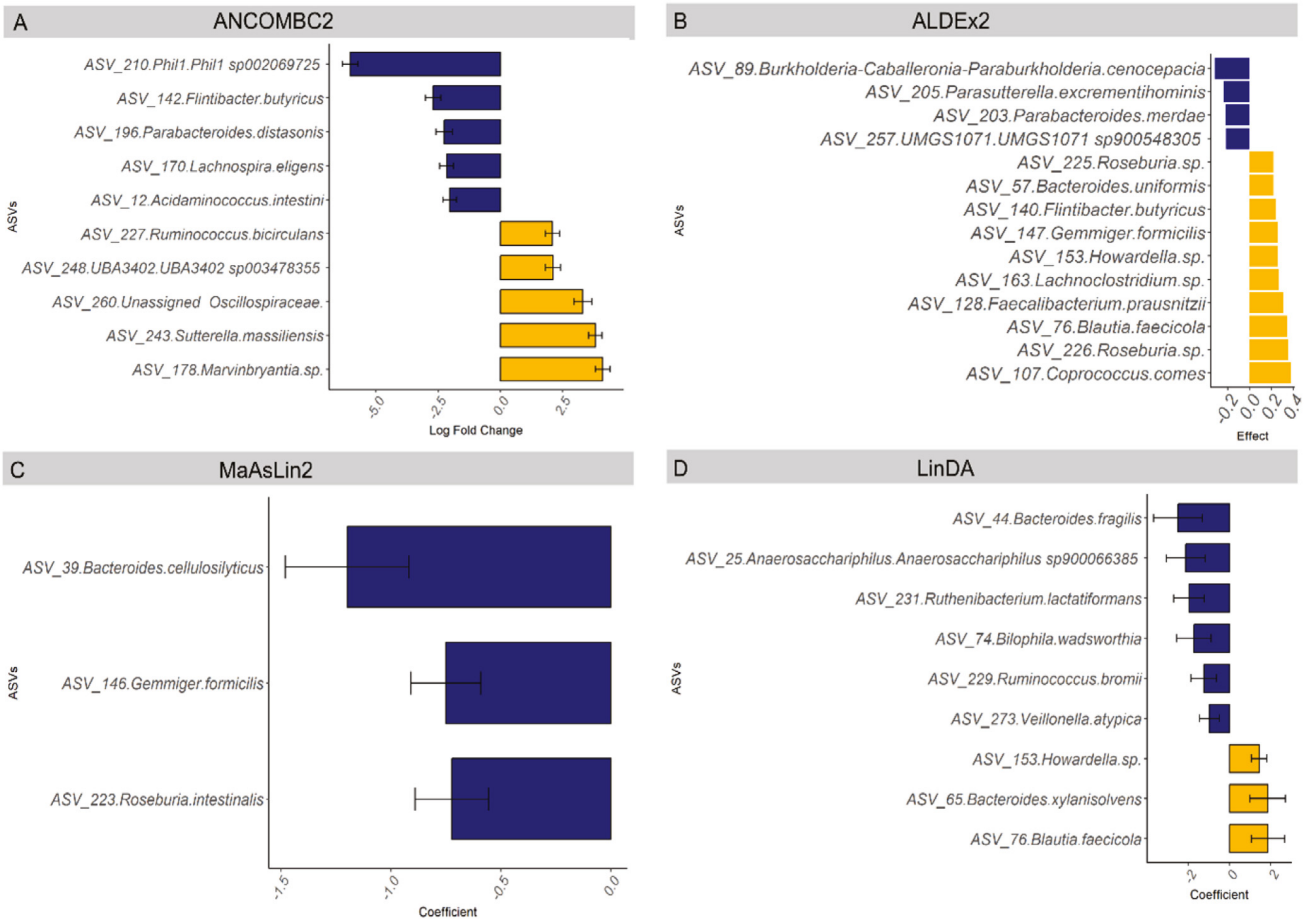
Microbial differential abundance comparing distal colon mucosa for adenoma and no-polyp participants. (A) ANCOMBC2 analysis (*P* < .05, log fold change >1.5) (B) ALDEx2 (effect size >0.15). (C) MaAsLin analysis (*P* < .05). (D) LinDA analysis (*P* < .05). Yellow, elevated in adenoma; blue, decreased in adenoma; black bars indicate standard error. All analyses include age, sex, and BMI as covariates, except for ALDEx2 which includes age and sex. (n = 29 adenoma, 52 control).

Figure 4 shows the differentially abundant ASVs in the distal colon from adenoma and no-polyp participants using the 4 models. The models were highly variable, although ALDEx2 and LinDA reported enrichment of *Blautia faecicola* and *Howardella sp*. from participants with polyps. Other ASVs shown to be differentially abundant were unique to the respective models.

**Figure 5:**
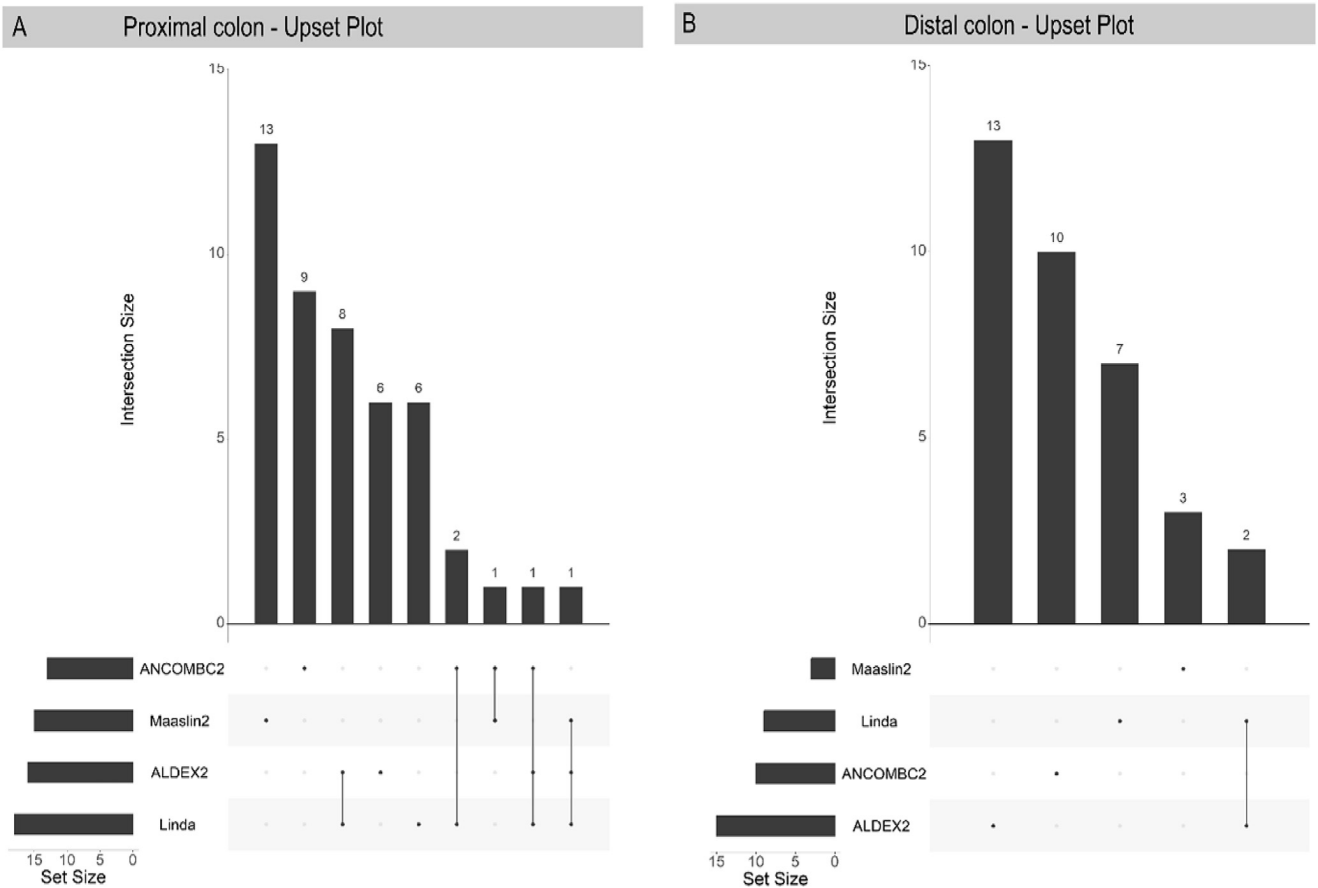
Upset Plots showing agreement between univariate differential abundance measures. (A) Proximal colon, (B) Distal colon. The plots include all ASVs that showed differential abundance between people with polyps and those without (ANCOMBC2 *P* < .05, log fold change >1.5; ALDEx2, effect size >0.15 ALDEx2, effect size >0.15; MaAsLin analysis, and LinDA analysis, *P* < .05).

To show similarities and differences between models an UpSet plot was used (Figure 5). ALDEx2 and LinDA modeling showed the most agreement, although there was considerable variability between models.

### Microbiota in Advanced and Nonadvanced Adenomas

While not the primary focus of our cohort design, we carried out microbiome analysis to compare the 16 specimens of advanced adenoma (defined as >10mm or tubulovillous histology) with the 52 non-advanced tubular adenoma (LGD <10mm) (Figures A1–A4). No differences were observed in alpha or beta diversity between these microbial communities. We applied the 4 bioinformatic models to investigate enrichment of individual ASVs. This revealed that different ASVs of unassigned *Lachnospiraceae* were enriched in advanced and non-advanced adenomas, suggesting the need to obtain even higher-resolution taxonomy methodologies to better understand these associations.

## Discussion

There are established mechanistic links between some pathogenic bacteria and CRC development, eg, enterotoxic *B fragilis* and pks + *E coli* [2]. Less is known about mucosal-resident microbiota located in close proximity to precancerous, adenomatous polyps. To better understand the relationship between gut microbiomes and early bowel neoplasia, here we used 16S rRNA taxonomy profiling of biopsied mucosa from the proximal and distal colon adjacent to LGD adenomas, which are early premalignant lesions, to compare with polyp-free control mucosa. From a microbiome diversity viewpoint, there was no significant dysbiosis in mucosal communities in patients with LGD adenoma compared to polyp-free participants. This finding is consistent with reports that suggested that differences in the polyp microbiome are subtle during the early precancerous stage.^43^^,^^44^ However, other studies reported statistically significant differences in alpha and beta diversity in differentiating participants with and without colonic polyps.^22^^,^^23^^,^^45^ In attempts to address bias, we applied 4 bioinformatic tools to measure differential microbial abundance, which identified select ASVs in both proximal and distal colons that separated adenoma patients from controls. Together, these findings suggest that the differences in mucosal microbiota in the early stages of adenoma development are subtle, and individual variation dominates microbiome heterogeneity between patient groups.

Our study identified microbiota that were previously found in a few studies that examined microbiota directly at the mucosa in participants with adenomas and healthy controls. ASVs associated with *Escherichia-Shigella*^25^ *Barnesiella*^26^ and *R gnavus*^27^ were elevated, and *Faecalibacterium*^21^ and *Klebsiella*^26^ depleted, in participants with polyps compared to those without. Differences in our results compared to these studies may be due to biases introduced by sequencing different regions of the 16S rRNA gene for taxonomy assignment (eg, V1–V2^23^ or V4 only^27^ compared to V3–V4 in this study) and differences in downstream bioinformatic pipelines that advance over time.

### Microbiota at the Proximal Colon Mucosa

To address the potential bias and heterogeneity introduced by bioinformatic models, we utilized 4 contemporary and emerging algorithms with covariates to detect differentially abundant microbes between sample groups. Our conservative analysis for reporting required differential detection in at least 2 of the bioinformatic methods, which revealed ASVs from *P distasonis, B uniformis, Escherichia-Shigella,* and *F periodonticum* were elevated in participants with early adenomas compared to those without polyps.

The literature on the relationship of particular microbes and CRC development is highly heterogeneous, in part mediated by different study designs, sample sizes, and bioinformatic analyses methods used, and this challenges interpretations. For example, the literature is dominated by microbiome taxonomy studies that analyze patient stool to infer gut microbiomes, but these studies lack the precision of colonoscopic-based sampling which enables direct analysis of microbes at the mucosal surface, rather than luminal communities.

In our study, we detected *P distasonis* to be elevated in patients with adenoma. Interestingly, this microorganism has previously been reported to suppress inflammatory cytokines, reduce protumorigenic AKT activation, and promote apoptosis in a mouse CRC model.^46^^,^^47^ However, other studies have reported increased *P distasonis* in stool samples from high-risk adenoma patients,^44^ and moreover, increased abundance in stool from adenoma to carcinoma patients,^48^ which is more in line with our study findings based on gut mucosa sampling. We observed *B uniformis* to be elevated in participants with adenoma compared to those without. While our findings are in the context of early bowel neoplasia, it agrees with a metagenomic analysis of 86 CRC and 86 matched controls from 6 projects that found *B uniformis* was a CRC-enriched microbe.^49^ Furthermore, evidence for the protumorigenic potential of *B uniformis* comes from a large population-based study showing increased risk of a CRC diagnosis following bacteremia with *B uniformis*.^50^ However, other studies have shown reduced fecal *B uniformis* in CRC patients compared to controls.^51^^,^^52^ Interestingly, *F periodonticum,* an oral commensurate closely related to *F nucleatum*, a microbe strongly implicated in colonic tumourigenesis,^53^, ^54^, ^55^ was also enriched in some participants with polyps in this study. *F periodonticum,* like *F nucleatum*, has the *FadA* gene, which produces a virulence factor that invades host cells and loosens cell-cell junctions.^56^

We found that some ASVs assigned to the *Lachnospiraceae* were consistently elevated in the proximal colon mucosa of adenoma patients. The family *Lachnospiraceae* encompasses a wide range of anerobic genera, many known to metabolize dietary fiber into short-chain fatty acids, which is generally thought to be cancer-protective. The use of short-read V3–V4 region sequencing adopted in this study limits the ability to further refine the taxonomy of involved *Lachnospiraceae*. Some *Lachnospiraceae (Blautia* and *Dorea)* and closely related *Oscillospiraceae* family members (*Ruminococcus*) have been shown to be enriched in normal colon tissue compared to CRC patient colon tissue.^57^ In contrast, we observed UCG-002 (*Oscillospiraceae*) to be elevated in patients with adenoma after we controlled for covariates. In support of this, *Lachnoclostridium spp.* (reassigned to *Clostridium*) are reportedly increased in stool samples from participants with adenomas compared to controls.^58^ *R gnavus* is an exemplar *Lachnospiraceae* family member (now *Oscillospiraceae)* that has previously been identified to be elevated in adenomas^27^ and is associated with inflammatory bowel disease.^59^^,^^60^ This is consistent with the idea that early adenomas may develop in an inflammatory microenvironment, promoted by certain microbes and proinflammatory cytokine fluxes.^61^

The only microbe detected in the majority of bioinformatic models to be depleted in participants with proximal adenoma was *Bacteroides/Phocaeicola massiliensis*. This result differs from previous studies, where CRC tumor tissue showed increased abundance of *B massiliensis* compared to 10 cm-adjacent normal tissue,^62^ and fecal samples from tubular adenoma patients^63^ and advanced adenoma and carcinoma patients^64^ showed increased abundance of *B massiliensis* compared to controls. However, *B massiliensis* is reportedly enriched in the fecal microbiome of responders to checkpoint inhibitors in various cancers,^65^ while another study reported that *B massiliensis* was enriched in CRC from patients with deficient mismatch repair, but also enriched in normal tissue in patients with proficient mismatch repair CRC,^66^ suggesting that the effect of *B massiliensis* is context dependent. *Anaerostipes hadrus* has also previously been shown to be depleted in stool samples of CRC patients.^67^^,^^68^ An analysis of 6 stool metagenomic CRC datasets also identified *Anaerostipes* species as depleted in CRC.^69^ This protective role may be due to its role as a butyrate producer, given that butyrate is associated with colon health.^70^

### Microbiota at the Distal Colon Mucosa

Participants with distal adenomas showed a different pattern of microbial abundance compared to those with proximal adenoma, and overall showed less agreement between differential abundance bioinformatic methods compared to the analyses of proximal located adenomas. There was too much variance amongst models to reliably identify depleted microbes in patients with distal adenoma. However, 2 of 4 univariate models showed elevations in ASVs associated with *Howardella* and *Blautia* in participants with polyps. *Blautia* is a member of the *Lachnospiraceae*, a family which we also found to be elevated in proximal adenoma from patients enriched with LGD adenomas. The genus *Howardella* is associated with both health and dysbiosis. It has been shown to be enriched in gastric cancers,^71^ in CRC mucosa compared to stool samples,^72^ and in rectal cancer compared to sigmoidal cancers.^73^

### Strengths and Limitations

While there are numerous studies examining the fecal microbiome from CRC patients, a strength of this study was to examine the gut mucosal microbiome in participants enriched with LGD colorectal adenomas as an early event in CRC carcinogenesis pathway. Mean participant age of cases was 64, which is similar to the mean age of CRC diagnosis. Moreover, we could examine the microbiome with spatial consideration of proximal and distal colonic locations. Another beneficial feature of our study is the control group was age-, sex-, and BMI-matched symptomatic colonoscopy patients, rather than healthy controls which may have different microbiome community structures. Aware of the limited consistency in studies detecting differentially abundant microbiota [105], we applied 4 bioinformatic tools with covariates striving to find consistently reported microbes. This was partially effective but also limiting the depth of our report to focus on the few commonly reported microorganisms found across the models. It is evident from our report that improvements are needed in bioinformatic models and this will be important to improve interstudy findings.

We sequenced the mucosal microbiome adjacent to bowel adenoma. It would have been ideal to study the neoplasm directly, however this could not be carried out in our study as the intact polyp was required for clinical diagnosis. Although our matched non-polyp cohort was controlled for sex, age, and BMI, we could not control for other variables, including diet that may partially explain the microbiome variation seen in this study. As biopsy specimens passed through the colonoscope channel, we cannot exclude the possibility of minor ASVs originating from elsewhere, including luminal contents. To counter this, specimens were rinsed prior to DNA extraction to remove major sources of nonmucosal contaminants.

## Conclusion

This study showed subtle gut microbiome differences in the proximal and distal mucosa from patients with LDG adenomas compared to colonoscopy-confirmed, polyp-negative patients. At a community level, differences were not readily evident, but specific ASVs could be detected by differential abundance mapping of taxa. Our study provides evidence that the gut microbiome is altered in the early stages of bowel neoplasia and establishes some leads for follow-up studies to pursue putative relationships with CRC carcinogenesis.

## Supplementary Materials

Figure A1

Figure A2

Figure A3

Figure A4

Table A1

Tables A2–A3
