# Supplementary figures and images for: Gut Mucosal Microbiome of Patients With Low-Grade Adenomatous Bowel Polyps
Source: Gastro Hep Adv. 2025 Apr 28;4(8):100687. doi: 10.1016/j.gastha.2025.100687 (PMC12171549; doi:10.1016/j.gastha.2025.100687)

Fig S1

A

Advanced

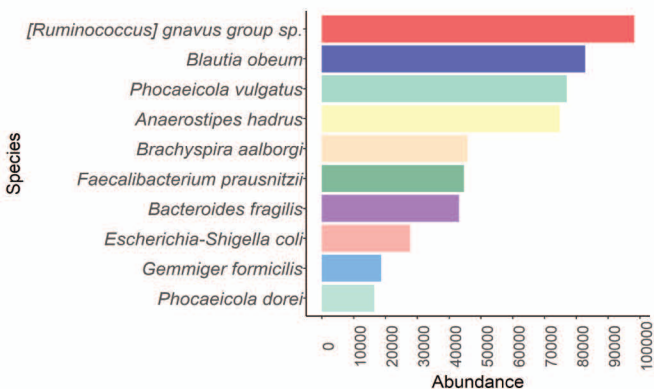

B

Non-advanced

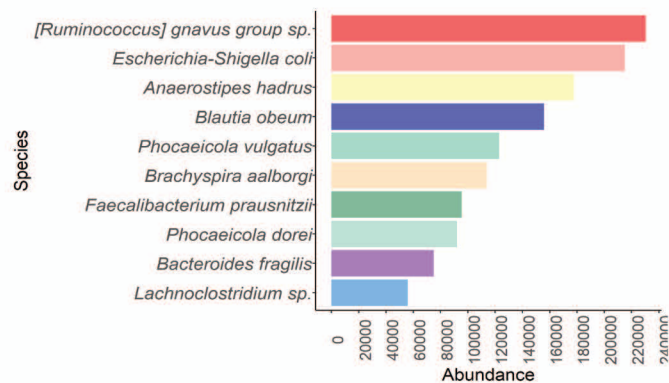

C

Advanced

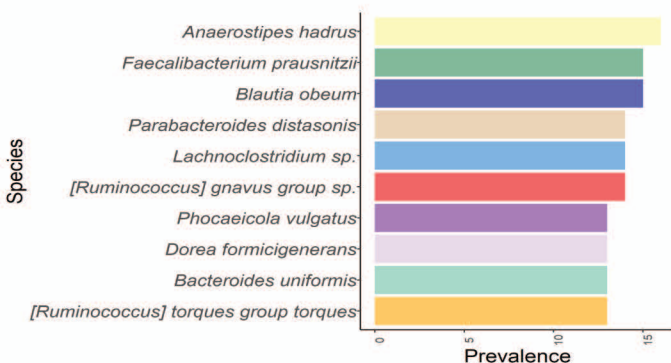

D

Non-advanced

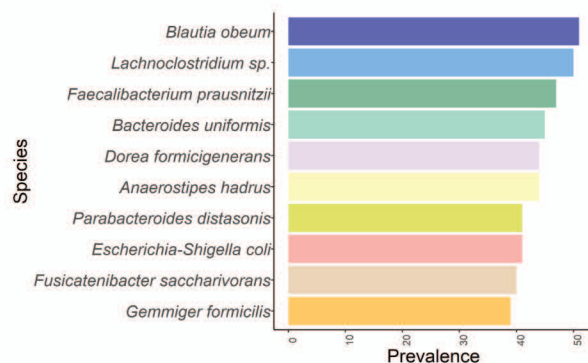

Supplement: Figure A1 [file mmc1.pdf]

Fig S2

A

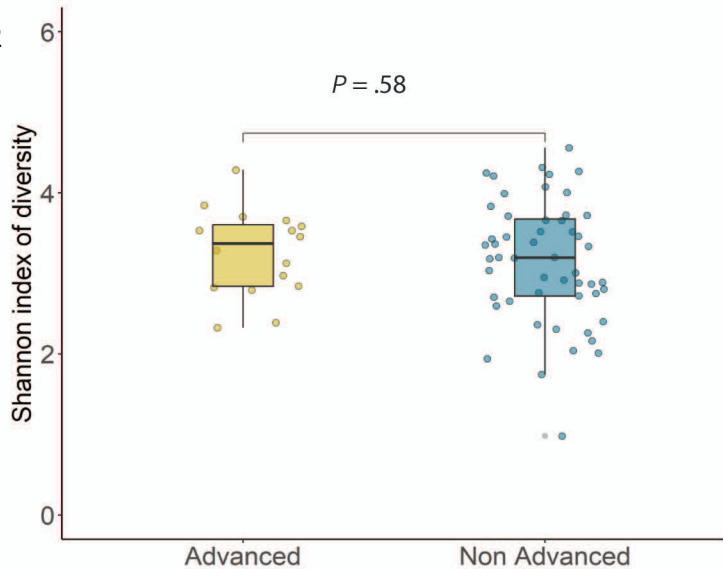

B

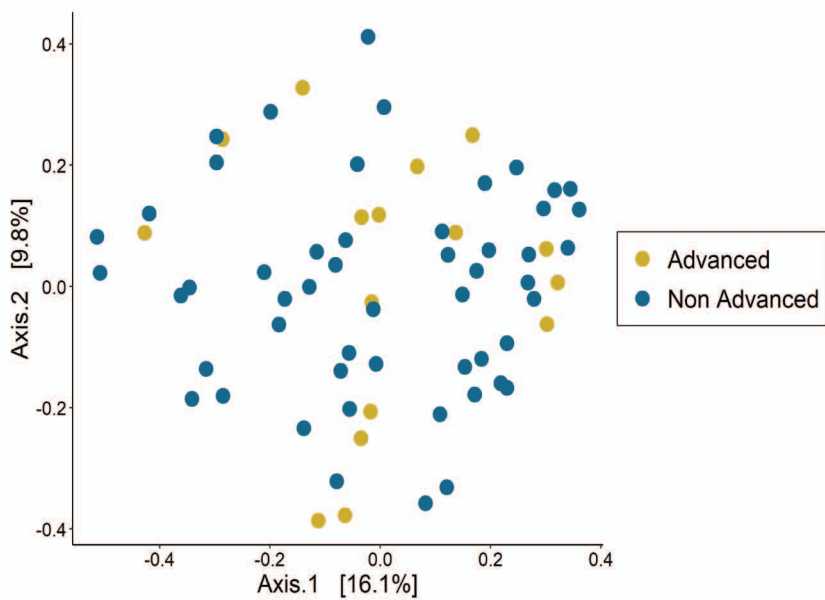

Supplement: Figure A2 [file mmc2.pdf]

Fig S3

ANCOMB2

A

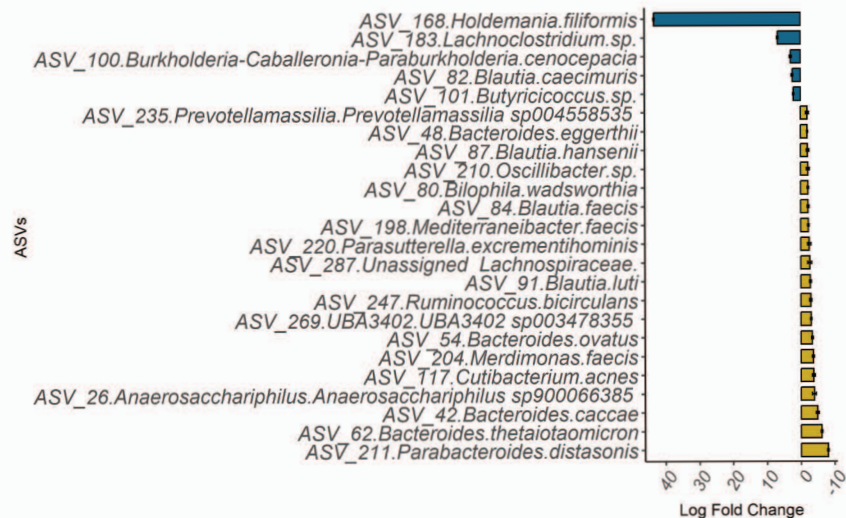

ALDEx2

B

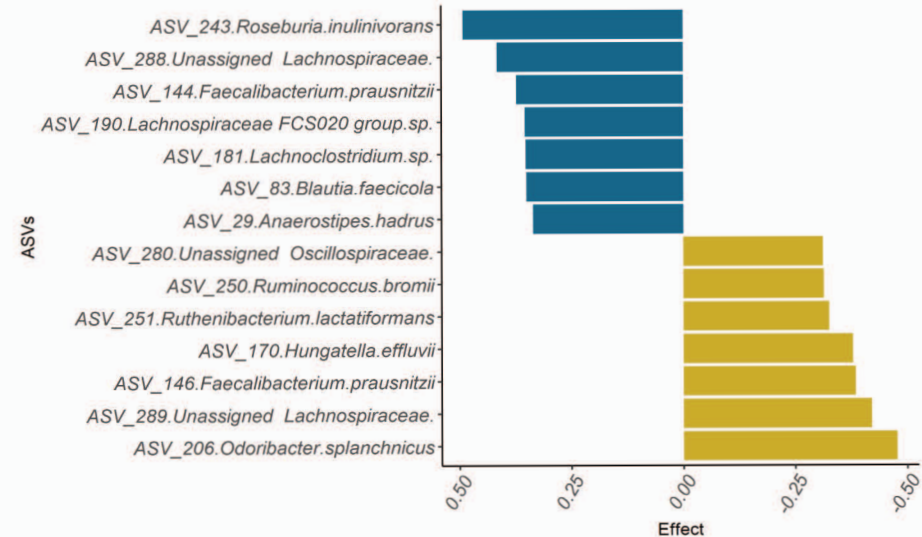

MaAsLin2

C

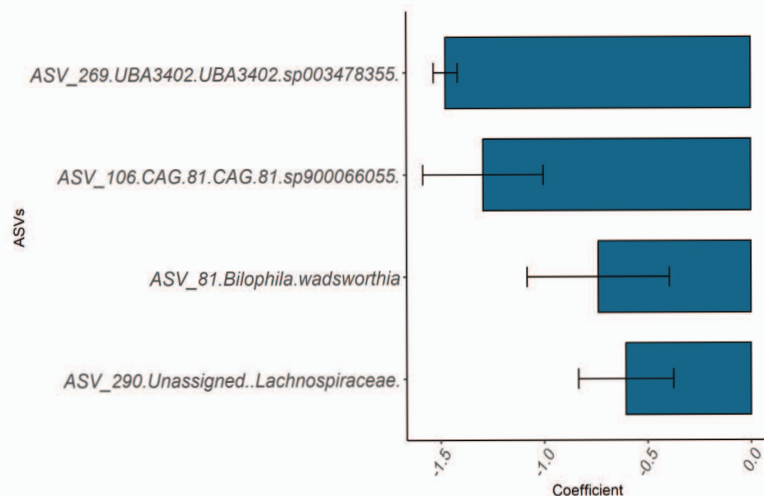

LinDA

D

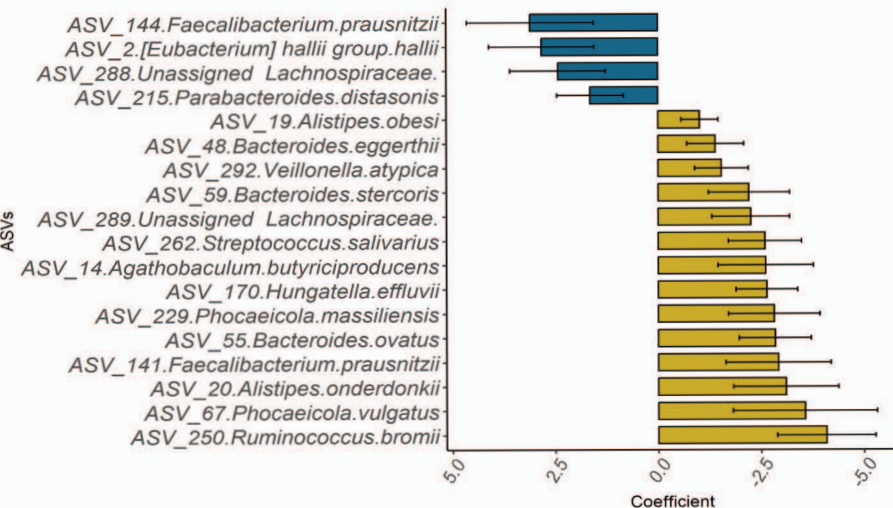

Supplement: Figure A3 [file mmc3.pdf]

Fig S4

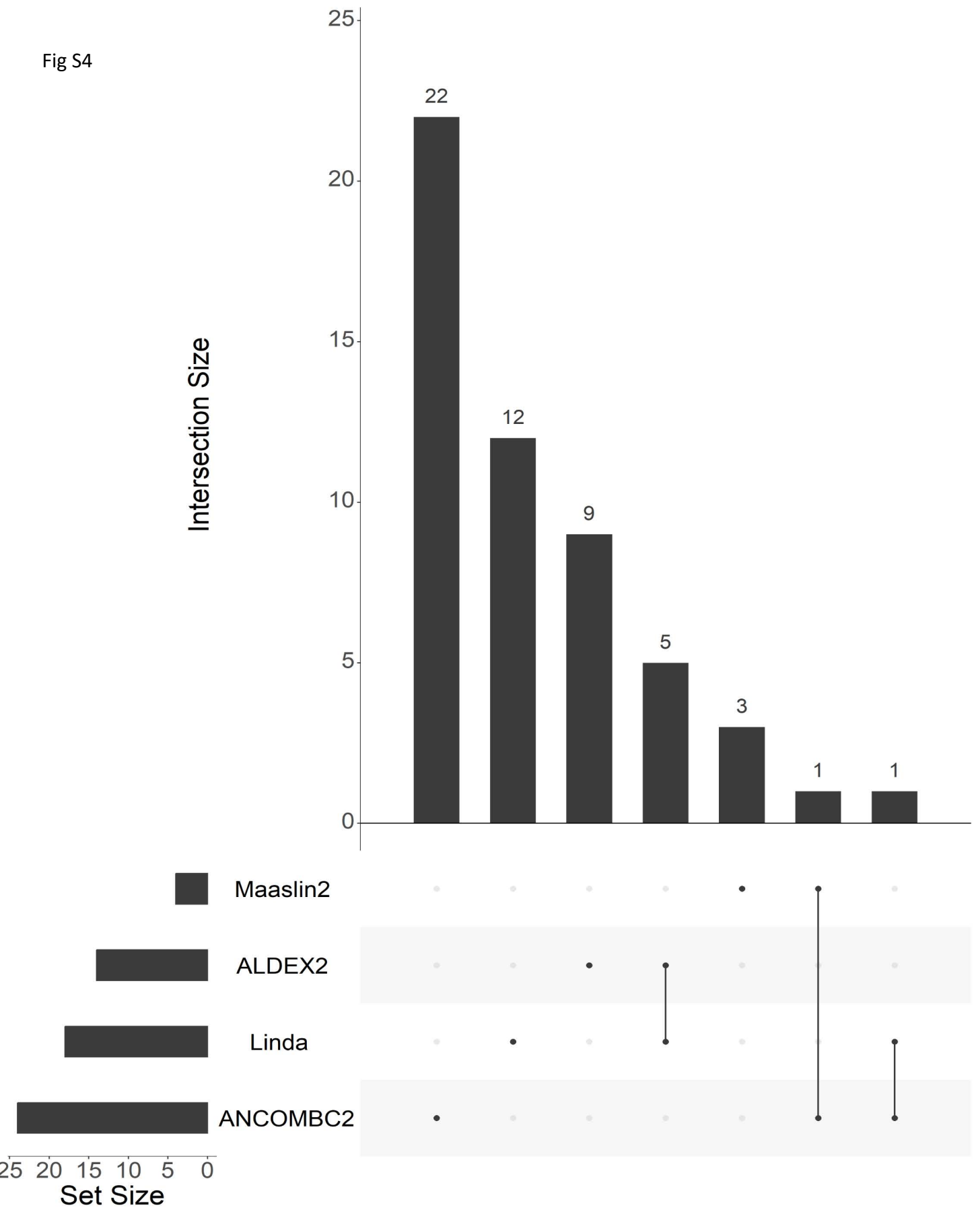

Supplement: Figure A4 [file mmc4.pdf]
