# Supplementary material for: Gut Mucosal Microbiome of Patients With Low-Grade Adenomatous Bowel Polyps
Source: Gastro Hep Adv. 2025 Apr 28;4(8):100687. doi: 10.1016/j.gastha.2025.100687 (PMC12171549; doi:10.1016/j.gastha.2025.100687)
Supplement: Tables A2–A3 [file mmc6.docx]

**Table S2: Relative abundances of proximal colon mucosa microbiota for participants with polyps and those without.**

|  | Relative abundance | | P-value |  | Relative abundance | | P-value |
| --- | --- | --- | --- | --- | --- | --- | --- |
| Family  Taxonomy | No Polyp | Polyp |  | Genera  Taxonomy | No Polyp | Polyp |  |
| *Lachnospiraceae* | 0.542 | 0.510 | 0.44 | *Other* | 0.150 | 0.152 | 0.98 |
| *Bacteroidaceae* | 0.145 | 0.178 | 0.31 | *Blautia* | 0.127 | 0.126 | 0.79 |
| *Oscillospiraceae* | 0.109 | 0.104 | 0.58 | *Anaerostipes* | 0.134 | 0.084 | 0.07 |
| *Enterobacteriaceae* | 0.053 | 0.086 | 0.47 | *Phocaeicola* | 0.106 | 0.106 | 0.95 |
| *Other* | 0.066 | 0.066 | 0.84 | ***Ruminococcus gnavus group*** | 0.060 | 0.091 | 0.026* |
| ***Sutterellaceae*** | 0.025 | 0.046 | 0.02* | *Escherichia-Shigella* | 0.052 | 0.086 | 0.47 |
| *Coriobacteriaceae* | 0.015 | 0.011 | 0.82 | *Faecalibacterium* | 0.060 | 0.066 | 0.20 |
| *Erysipelotrichaceae* | 0.016 | 0.005 | 0.12 | *Bacteroides* | 0.038 | 0.070 | 0.15 |
| *Bifidobacteriaceae* | 0.009 | 0.008 | 0.73 | *Mediterraneibacter* | 0.045 | 0.043 | 0.25 |
| *Clostridiaceae* | 0.003 | 0.011 | 0.70 | *Ruminococcus torques group* | 0.030 | 0.030 | 0.71 |
| *Akkermansiaceae* | 0.004 | 0.005 | 0.07 | *Lachnoclostridium* | 0.021 | 0.023 | 0.98 |
| *Lactobacillaceae* | 0.005 | 0.004 | 0.34 | *Gemmiger* | 0.023 | 0.020 | 0.83 |
| *Family XI* | 0.009 | 0.000 | 0.19 | *Roseburia* | 0.021 | 0.017 | 0.48 |
|  |  |  |  | *Sutterella* | 0.020 | 0.010 | 0.06 |
| P-value = Wilcoxon test | | | | | | | |

**Table S3: Relative abundances of distal colon mucosa microbiota for participants with polyps and those without**.

| Family  Taxonomy | Relative abundance | | P-value | Genera | Relative abundance | | P-value |
| --- | --- | --- | --- | --- | --- | --- | --- |
|  | No Polyp | Polyp |  | Taxonomy | No Polyp | Polyp |  |
| *Lachnospiraceae* | 0.533 | 0.515 | 0.74 | *Other* | 0.149 | 0.153 | 0.91 |
| *Bacteroidaceae* | 0.158 | 0.159 | 0.72 | *Blautia* | 0.134 | 0.133 | 0.73 |
| *Oscillospiraceae* | 0.107 | 0.081 | 0.3 | *Phocaeicola* | 0.100 | 0.102 | 0.36 |
| *Enterobacteriaceae* | 0.074 | 0.094 | 0.39 | *Anaerostipes* | 0.102 | 0.094 | 0.67 |
| *Other* | 0.063 | 0.055 | 0.12 | *Escherichia-Shigella* | 0.074 | 0.093 | 0.34 |
| ***Akkermansiaceae*** | 0.008 | 0.020 | 0.03* | *Ruminococcus] gnavus group* | 0.079 | 0.083 | 0.92 |
| *Acidaminococcaceae* | 0.014 | 0.013 | 0.98 | *Bacteroides* | 0.057 | 0.056 | 0.82 |
| *Coriobacteriaceae* | 0.011 | 0.011 | 0.84 | *Faecalibacterium* | 0.05 | 0.037 | 0.89 |
| ***Erysipelotrichaceae*** | 0.006 | 0.012 | 0.045* | *Lachnoclostridium* | 0.043 | 0.036 | 0.96 |
| *Bifidobacteriaceae* | 0.007 | 0.012 | 0.68 | *Ruminococcus torques group* | 0.031 | 0.037 | 0.94 |
| *Peptostreptococcaceae* | 0.008 | 0.009 | 0.28 | *Gemmiger* | 0.031 | 0.024 | 0.66 |
| *Sutterellaceae* | 0.007 | 0.009 | 0.53 | *Mediterraneibacter* | 0.035 | 0.024 | 0.92 |
| *Streptococcaceae* | 0.004 | 0.007 | 0.15 | *Roseburia* | 0.023 | 0.015 | 0.99 |
|  |  |  |  | ***Akkermansia*** | 0.009 | 0.020 | 0.029* |
| P-value = Wilcoxon test | | | | | | | |
