# Supplementary material for: Gut Mucosal Microbiome of Patients With Low-Grade Adenomatous Bowel Polyps
Source: Gastro Hep Adv. 2025 Apr 28;4(8):100687. doi: 10.1016/j.gastha.2025.100687 (PMC12171549; doi:10.1016/j.gastha.2025.100687)
Supplement: Supplementary Materials [file mmc7.pdf]

Supplementary Materials Summary

Gut mucosal microbiome of patients with low-grade adenomatous bowel polyps

Authors: Zoe Welham[1], Jun Li[1], Benita Tse[1], Alexander Engel[2,3], Mark P. Molloy[1*]

1 Bowel Cancer and Biomarker Laboratory, Kolling Institute, School of Medical Sciences, The University of Sydney, St. Leonards, Australia 2065

2 Colorectal Surgical Unit, Royal North Shore Hospital, St. Leonards, Australia, 2065.

3 Sydney Medical School, The University of Sydney, Sydney, Australia 2006.

Figure S1 - Abundance and prevalence of microbiota for advanced (tubulovillous adenoma or >10mm) and non-advanced (tubular adenoma <10mm) bowel polyps. Top 10 microbial species for Cumulative abundance (A,C) and Prevalence (B,D).

Figure S2- Mucosal microbiome diversity for advanced (Gold) and non-advanced adenomas (Blue) based on alpha diversity using Shannon Index (A) and beta diversity using Principal Coordinates Analysis to display Bray-Curtis dissimilarity score.

Figure S3 - Microbial differential abundance comparing advanced and non-advanced adenoma. A. ANCOMBC2 analysis ($P < .05$, LFC > 1.5) B. ALDEx2 (effect size > 0.15). C. MaAsLin2 ($P < .05$). D. LinDA analysis ($P < .05$). Yellow, elevated in advanced adenoma; Blue, elevated in non-advanced adenoma; Black bars indicate standard error. All analyses include age, sex and BMI as covariates, except for ALDEx2 which includes age and sex. (n= 16 advanced adenoma, 52 non-advanced adenoma).

Figure S4 - Upset Plots showing agreement between four univariate differential abundance methods. The plots include all ASVs that showed differential abundance between advanced and non-advanced adenoma.

Table S1 – Participant and specimen details

Table S2 - Relative abundances of proximal colon mucosa microbiota for participants with polyps and those without.

Table S3 - Relative abundances of distal colon mucosa microbiota for participants with polyps and those without.
